# Exploring the Behavior of Users With Attention-Deficit/Hyperactivity Disorder on Twitter: Comparative Analysis of Tweet Content and User Interactions

**DOI:** 10.2196/43439

**Published:** 2023-05-17

**Authors:** Liuliu Chen, Jiwon Jeong, Bridgette Simpkins, Emilio Ferrara

**Affiliations:** 1 Department of Electrical and Computer Engineering Viterbi School of Engineering University of Southern California Los Angeles, CA United States; 2 Department of Computer Science Viterbi School of Engineering University of Southern California Los Angeles, CA United States; 3 Annenberg School for Communication and Journalism University of Southern California Los Angeles, CA United States

**Keywords:** social media, mental health, attention-deficit/hyperactivity disorder, ADHD, Twitter, behaviors, interactions

## Abstract

**Background:**

With the widespread use of social media, people share their real-time thoughts and feelings via interactions on these platforms, including those revolving around mental health problems. This can provide a new opportunity for researchers to collect health-related data to study and analyze mental disorders. However, as one of the most common mental disorders, there are few studies regarding the manifestations of attention-deficit/hyperactivity disorder (ADHD) on social media.

**Objective:**

This study aims to examine and identify the different behavioral patterns and interactions of users with ADHD on Twitter through the text content and metadata of their posted tweets.

**Methods:**

First, we built 2 data sets: an ADHD user data set containing 3135 users who explicitly reported having ADHD on Twitter and a control data set made up of 3223 randomly selected Twitter users without ADHD. All historical tweets of users in both data sets were collected. We applied mixed methods in this study. We performed Top2Vec topic modeling to extract topics frequently mentioned by users with ADHD and those without ADHD and used thematic analysis to further compare the differences in contents that were discussed by the 2 groups under these topics. We used a distillBERT sentiment analysis model to calculate the sentiment scores for the emotion categories and compared the sentiment intensity and frequency. Finally, we extracted users’ posting time, tweet categories, and the number of followers and followings from the metadata of tweets and compared the statistical distribution of these features between ADHD and non-ADHD groups.

**Results:**

In contrast to the control group of the non-ADHD data set, users with ADHD tweeted about the inability to concentrate and manage time, sleep disturbance, and drug abuse. Users with ADHD felt confusion and annoyance more frequently, while they felt less excitement, caring, and curiosity (all *P*<.001). Users with ADHD were more sensitive to emotions and felt more intense feelings of nervousness, sadness, confusion, anger, and amusement (all *P*<.001). As for the posting characteristics, compared with controls, users with ADHD were more active in posting tweets (*P*=.04), especially at night between midnight and 6 AM (*P*<.001); posting more tweets with original content (*P*<.001); and following fewer people on Twitter (*P*<.001).

**Conclusions:**

This study revealed how users with ADHD behave and interact differently on Twitter compared with those without ADHD. On the basis of these differences, researchers, psychiatrists, and clinicians can use Twitter as a potentially powerful platform to monitor and study people with ADHD, provide additional health care support to them, improve the diagnostic criteria of ADHD, and design complementary tools for automatic ADHD detection.

## Introduction

### Background

Mental health has become a major issue that demands continuous attention, as it causes individuals to have other difficulties [1]. The Centers for Disease Control and Prevention (CDC) states that attention-deficit/hyperactivity disorder (ADHD) “is one of the most common neurodevelopmental disorders of childhood” [2] that is frequently maintained until adulthood. Several studies emphasize the concern regarding ADHD. BlueCross BlueShield announced that in 2019, ADHD was found to be the second most impactful condition affecting people’s health in the United States [3]. The World Federation of ADHD International Consensus Statement has listed 8 distresses associated with ADHD, including quality of life, emotional and social impairment, premature death and suicide, educational underachievement, and more [4].

The diagnosis of ADHD is complex because of the blurred boundary between the affected and unaffected status. The Diagnostic and Statistical Manual of Mental Disorders, Fifth Edition (DSM-5) provides guidelines for diagnostic standards and identifies 3 main patterns that people with ADHD present: inattention, hyperactivity, and impulsivity [5]. However, the manifestations of symptoms of ADHD may vary between children and adults. As children with ADHD grow into adulthood, their symptoms may be masked or develop into new conditions. ADHD has traditionally been observed and perceived as a disorder in children, leading to a lack of diagnoses in adults. Adults and children typically present the symptoms of inattention, hyperactivity, and impulsivity in different patterns [6]. Although DSM-5 updated the examples of symptoms for adults (aged ≥17 years) and changed the age of onset from age 7 to age 12 to better define how ADHD manifests in adulthood, many adults still face difficulty recalling their memory before age 12 and fitting their behaviors into diagnostic criteria. In addition, there is no single test for ADHD, and current ADHD assessments are subjective to psychiatrists’ experiences and patients’ self-reports [7]. In these situations, researchers, clinicians, and patients demand improved ADHD diagnostic criteria in DSM-5 and novel and objective assessment technology.

Social media platforms such as Twitter are gaining more popularity today. As of the fourth quarter of 2020, Twitter’s monetizable daily active users reached 192 million [8], and as of April 2021, 93.4% of Twitter users worldwide were aged ≥18 years [9]. Previous studies corroborate that Twitter provides a safe and strong environment, allowing people to discuss their mental health problems, seek support, and connect with communities [10]. People are also more likely to share their honest thoughts and opinions because of the user anonymity of Twitter. Compared with the consultation diagnosis process that relies on consultees’ memories, Twitter records users’ feelings and activities instantaneously, which might reflect ADHD symptoms more accurately. Moreover, the temporal information and metadata provided by Twitter offer unique behavioral insights to explore, such as the distribution of ADHD users’ posts through the hour of the day and users’ preference for posting original content or retweeting. Examining the social media habits of individuals with ADHD may enable a deeper understanding of the behaviors and emotions related to ADHD [11,12].

Some previous studies have already investigated the representation of mental illness on Twitter, including depression [13,14], posttraumatic stress disorder (PTSD) [15,16], and bipolar disorder [17]. However, only a few studies have focused on ADHD [11,18-20]. Existing analyses primarily focus on the language features of ADHD on Twitter [11,18]. Previously, researchers analyzed how ADHD users construct their sentences and identified their preferences for using first-person narrative and negative-tone words in tweets [18]. Other scholars conducted a case study to explore ADHD users’ concerns about their disorder [19]. However, it is still incomprehensive and unclear as to how ADHD users behave and interact on Twitter.

### Objectives

This study aimed to uncover the patterns of behaviors and interactions of users with ADHD on Twitter. Specifically, we investigated the difference between Twitter users with ADHD and Twitter users without ADHD based on the following three aspects: (1) the pattern of talking about different topics; (2) the pattern of expressing emotions; and (3) the interactions of time, tweet type, followers, and followings.

## Methods

### Data Collection

To create the ADHD data set, we used the Twitter application programming interface to search for self-identified users who tweeted in English. A user was identified as a patient with ADHD based on the following statements in their original tweets (not retweets and quoted tweets): “I have/am ADHD” and “I was diagnosed with ADHD.” Approximately 5000 users were identified and subsequently filtered through regular expressions to remove users who suspected they have ADHD or were in the process of being diagnosed. Finally, the 3 researchers independently annotated and verified the authenticity of all self-proclaimed users by manually reviewing the context of the tweets that contained the self-reporting phrase, and those with mutual agreement were retained (Cohen κ=1.0). From the remaining 3135 users with ADHD, we collected all historical tweets and returned 8.3 million (n=8,300,768) tweets from April 28, 2009, to March 24, 2022, consisting of original tweets, replies, retweets, and quoted tweets.

**Figure 1:**
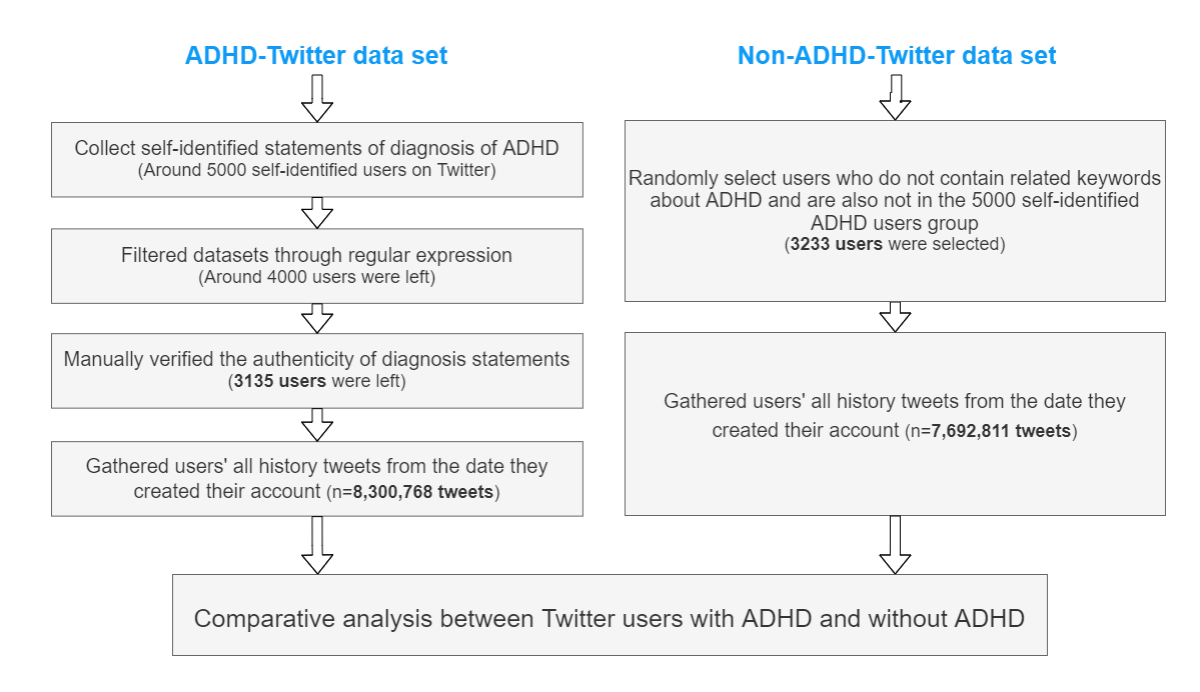
Flowchart of data collection procedures. ADHD: attention-deficit/hyperactivity disorder.

A control group of 3223 (assumed) neurotypical (non-ADHD) users was formed for comparison. We randomly selected 5000 Twitter users who tweeted in real-time streaming without ADHD-related terms. After excluding commercial users’ and organizations’ accounts and verifying that these users were not in the ADHD data set, 3223 users remained. We cannot be certain that all these users are not affected by ADHD even if they did not report it in their tweets. It is likely that 3% to 9% of these users without ADHD have ADHD, given the rate of ADHD in the population [21]. This contamination will only obscure the differences between the 2 groups, and we make no attempt to remedy this. We downloaded all historical tweets for the 3223 users without ADHD and returned 7.6 million (n=7,692,811) tweets from April 6, 2009, to March 25, 2022. The collection procedures are illustrated in Figure 1.

Our final data contain 2 data sets: the ADHD data set (users=3135; tweets=8,300,768) and the non-ADHD data set (users=3223; tweets=7,692,811). Tweets in both data sets contained text content, posted time, and language and were classified into 1 of 4 categories: original tweets, retweeted tweets, replied tweets, and quoted tweets. Original tweets are content entirely generated by users without referencing other tweets [22]. A retweet is a reposting tweet without any originality [22]. Replied tweets are responses to other users’ tweets, and quoted tweets allow users to append their comments to other people’s tweets [23]. In addition to the user-posted tweets, both data sets contain users’ metadata such as self-defined locations, descriptions of profiles, the number of followers and followings, and more.

### Data Preprocessing

For text-based analysis, only the original tweets were used (ADHD: 2.2 million; non-ADHD: 1.3 million). We preprocessed the text in 3 stages: data cleaning, stop words removal, and tokenization and lemmatization.

- Data cleaning: first, we converted all text to lower case for easier handling. Digits, URLs, and punctuation were removed. Special characters that frequently appear on social media, such as hashtags (#), emojis, and reply signs (@reply), were also removed. This resulted in some tweets being empty or having ≤3 words, which were discarded.
- Stop word removal: in this step, we customized a list of stop words comprising pronouns, conjunctions, and abbreviated words based on the domain knowledge of ADHD and Twitter. As these words carry little valuable information, eliminating them helps expose substantial clues in the remaining texts.
- Tokenization and lemmatization: to reduce the repeated character sequence and better adapt the language used on Twitter, we applied the Natural Language Toolkit’s (NLTK) TweetTokenizer [24] to split the text into tokens. Lemmatization with NLTK’s WordNetLemmatizer [24] was adopted instead of stemming, enabling us to deduce the meanings of tokens more precisely without discarding a large set of tokens because of shallow, inaccurate root-based filtering.

### Data Analysis

#### Topic Modeling

We aimed to detect and analyze the topics that users with ADHD and those without ADHD frequently interact with, so topic modeling was applied. Latent Dirichlet Allocation and Latent Semantic Analysis are common methods for topic modeling, but they have some limitations: (1) the number of topics is fixed, and it is difficult to find an optimal number; (2) the models cannot capture correlations between topics; and (3) some uninformative words were clustered as topics. To overcome these limitations, we trained a Top2Vec model [25]. Owing to the leverage of joint document and word semantic embedding, Top2Vec allowed us to automatically capture informative and representative topics and the number of topics in short text and to search similar topics and documents by keywords [25].

We created a list of ADHD-related keywords (Multimedia Appendix 1), such as “sleep” and “focus,” and programmed to use the built-in search function of Top2Vec model to get similar topics and related tweets in both ADHD and non-ADHD groups. We inspected the most frequently mentioned words in different topics and further investigated the text content under these topics. For each topic, we selected the top 10 text clusters with the highest cosine similarity to the queried topic keywords. We retrieved the 50 most frequently used words from the model in each cluster to generate word clouds for better visualization. For a more qualitative inspection, we reviewed the 100 relevant tweets that were most semantically similar to the topic in each cluster. The 3 researchers independently read these relevant tweets to summarize the content and emotions in each tweet. The researchers then discussed the summarization and inferences of each tweet until consensus was reached.

#### Sentiment Classification

To compare how different the sentiments expressed on Twitter by users with ADHD and those without ADHD are, we calculated sentiment scores using a pretrained DistillBERT model [26] from Huggingface. This model was trained on the GoEmotions data set [27], a robust corpus of Reddit comments with 27 human-annotated emotion categories published by Google Research. On the basis of the recent psychological concepts and methodologies to capture the more complex “semantic space” of emotions by analyzing the distribution of emotional responses [28], researchers have discovered 27 distinct types of emotional experiences conveyed by different means [29-33], such as facial expression and speech prosody. These methods and findings were used in the GoEmotions study to create a refined taxonomy for text-based sentiment recognition. Twitter and Reddit are popular social media platforms that aggregate information into posts. Comments in GoEmotions are relatively short with a maximum word count of 30, whereas 91% of text in the ADHD data set and 94% of text in the non-ADHD data set are equal to or less than 30 words in length, with a maximum word count of 240. Therefore, this model is compatible with our ADHD-Twitter and non-ADHD–Twitter data sets.

The DistillBERT sentiment model generates a separate score for each emotion category, and the sum of the scores of all emotion categories equals 1. The rated text was later classified as the emotion category with the highest score. We classified the emotion category for each tweet from every user with an emotion score. Similar to other studies [34,35], we used the sentiment score to indicate the emotion intensity (ie, a higher sentiment score represents a stronger intensity for the emotion category) and calculated the number of tweets classified into each emotion category as the emotion frequency. We further compared the intensity and frequency of each emotion category between the 2 groups.

#### Twitter Interaction Features

We extracted several behavioral features related to Twitter interactions to determine the differences between the 2 data sets. The profiles of Twitter users include valuable metadata for us to explore users’ behavioral patterns such as the number of followers, followings, self-defined location, and many others. We are also interested in the preferred activity time of ADHD users and how they interact with Twitter by posting their original content or retweeting others’ opinions. All extracted features are listed in Table 1.

When extracting the features of the posting hour, we first inferred the geolocation for all users, because Twitter only provides a Unix Timestamp. We predict users’ countries and states (if they are from the United States) from their self-defined locations or directly from the coordinates provided by tweets, exclude unpredictable users, and convert the timestamp of a tweet to their exact local time.

**Table 1:** Twitter interaction features and description.

Behavioral feature	Description
Posting hour	The number of tweets per hour as a percentage of the total number of tweets for the day
Posting day	Number of posting tweets per day
Original ratio	The number of original tweets as a percentage of all tweets per user
Retweeted ratio	The number of retweeted tweets as a percentage of all tweets per user
Followers	The number of followers for each user
Followings	The number of people that the user is following for each user

### Statistical Analysis

Multiple statistical tests were performed to determine the significant differences in results from previous analyses. For sentiment classification and most behavioral features, the results of ADHD and non-ADHD data sets are independent; they have an approximately normal distribution, and their variances are homogeneous, so ANOVA was applied. Some behavioral features failed to satisfy the aforementioned conditions, in which case the nonparametric Mann-Whitney *U* test was used. Conventionally, the threshold for differentiating significant results from nonsignificant results is α=.05.

### Ethical Considerations

Conducting ADHD-related research using Twitter data raises important ethical and legal implications. In this study, we used the official Twitter application programming interface to collect only publicly available tweets that everyone could access without previous approval. According to Twitter’s privacy policy [36], when using Twitter, users have consented to the collection, storage, and use of these publicly available data for noncommercial research purposes. During the analysis, we followed Twitter’s developer agreement and policy [37]. All analyses were conducted in an aggregated data format, and geolocation information was inferred and used in conjunction with the attached Twitter content rather than on a stand-alone basis. No other sensitive data were derived or inferred from individual Twitter users. To further protect user privacy and reduce the risk of identifying individual Twitter users, we removed all usernames and identifiable details from the data.

## Results

### Topic Modeling

#### Topics Overview

The Top2Vec model automatically detected 1579 ADHD user clusters and 1197 non-ADHD user clusters. Clusters represent topics that users might discuss, and there may be associations and similarities between these topics. We manually checked the 10 clusters with the most users, resulting in 4 topics as follows that substantially differentiated between ADHD-Twitter and non-ADHD–Twitter data sets: concentration ability, time management, sleep issues, and drug abuse. Table 2 provides an overview of the topics.

**Table 2:** Topics and descriptions.

Topics	Description	Example keywords used for search	Number of tweets on ADHD^a^	Number of tweets on non-ADHD
Concentration ability	Whether users are capable of focusing on a task	Focus, focused, task, attention, productive	21,340	23,109
Time management	How users organize and plan to split time between different tasks	Priority, organize, prioritize, planning	16,541	12,280
Sleeping issues	Irregular sleep-awake rhythm	Sleep, insomnia, restless, awake, sleepy	15,850	8483
Drug abuse	Addiction to drugs	Drug, addiction, meth, smoking, nicotine	10,190	9632

^a^ADHD: attention-deficit/hyperactivity disorder.

#### Topic 1: Concentration Ability

Concentration ability defines whether people can focus on tasks for a prolonged time. We searched keywords such as “focus” and “tasks” to find what users discussed about this ability in both data sets. The Table 3 shows the top 15 most frequently mentioned words that highly correlated with this topic. The whole word list correlated to this topic is shown in Multimedia Appendix 1.

ADHD users frequently tweeted that ADHD makes them unable to concentrate on their tasks, they find starting tasks difficult, they are easily distracted, and they procrastinate to finish. Example tweets are as follow:

I actually cannot focus on a single thing right now pls help.

I cannot focus on work.

I don’t know about you but I need at least 6 hours to do a task that will take 20 minutes.

However, some ADHD users mentioned “hyperfixation” and “hyperfocus,” indicating they can also find themselves on the other extreme side of the focus spectrum:

I yell, hyperfocusing on a videogame for 8 hours straight.

...how passionate and excited I could be about my favorite things...that I was just ADH hyperfixation baby!

Users without ADHD referred to this topic more broadly and positively. Unlike users with ADHD, who mentioned their inability to focus, users without ADHD often described the activities they paid attention to and how they focused on various activities:

I love teruyama...focusing my content on YT will be the best thing to do.

...Its wild as hell how my two games I’ve been focusing on...

The live music was amazing, I was following the flow of guitar and paying no attention to anyone else. Must listen live at...

**Table 3:** Top 15 most frequently mentioned words related to concentration abilities among users with attention-deficit/hyperactivity disorder (ADHD) and users without ADHD.

ADHD group	Non-ADHD group
Productive	Attention
Focusing	Focused
Distracted	Focusing
Exhausted	Paying
Focus	Hollywood
Fixation	Teruyama
Symptom	Geometry
Fatigue	Listenlive
Mindfulness	Role
Motivation	Friends
Hyperfixation	Focus
Distraction	Impress
Fixated	Cityofmoncton
Struggle	Ancestry
Task	Span

#### Topic 2: Time Management

Some tweet clusters indicated that many users with ADHD struggled with time management. Table 4 shows that users with ADHD frequently described their inability to organize and complete tasks promptly using negative words such as “dysfunction,” “weakness,” and “hard”:

...Prioritization and time management are still my biggest weaknesses and I’m trying my best to fix it.

...It’s fxxking hard to make something a priority.

Users with ADHD often felt unmotivated to perform tasks outside of their interests. Moreover, they felt overwhelmed by tasks that disabled them from prioritizing tasks, leading to procrastination and poor time management:

...I get overwhelmed with information I can’t figure out how to organize and it just swims around in my head for days and days until everything clicks into place.

...I am so unmotivated when it comes to cleaning up and do not enjoy it. It is hard because I am not an organized person.

Regarding time management, users without ADHD mentioned work-oriented or political tasks and objects. On the other hand, ADHD users’ perspective on time management appeared more negative using words such as dysfunction, disability, and weakness often in relation to productivity and completing mundane tasks such as cleaning or organizing. The whole word list correlated to this topic is shown in Multimedia Appendix 1.

**Table 4:** Top 15 most frequently mentioned words related to time management among users with attention-deficit/hyperactivity disorder (ADHD) and users without ADHD.

ADHD group	Non-ADHD group
Prioritize	Ballot
Organize	Workshop
Organized	Folder
Dysfunction	Voter
Weakness	Election
Unmotivated	Linked
Organizing	Fujimoto
Task	Learning
Priority	Files
Strength	Voting
Clean	Organizing
Performative	Booklet_design
Resource	Vote
Workspace	Theatre
Hard	Document

#### Topic 3: Sleep Issues

Sleeping issues are some of the most impactful problems today. People with insomnia struggle to sleep at night or constantly feel sleepy during the daytime, negatively affecting their quality of life and exacerbating mental health problems [38,39]. As shown in Table 5, many users with ADHD struggle with insomnia and express restlessness and the need to use melatonin to sleep at night:

...I can’t sleep because I have restless hands wtf I want to cry n scream.

...I have insomnia due to my ADHD, so I take 3 mg of melatonin supplements almost every night...

The lack of sleep caused users with ADHD to feel exhausted, frustrated, anxious, and unable to focus:

...I’m so tired, I haven’t really slept in a week, and I think my brain is immune to melatonin.

...Insomnia I can cope with once in a while. A full week of it and I am genuinely starting to think death would be preferable.

On the other hand, users without ADHD seemed to have healthier, regulated sleep patterns. The topic of sleep appears in tweets about their routine, the use of alarms and snooze buttons, how they feel, and what they do when they wake up. The whole word list correlated to this topic is shown in Multimedia Appendix 1.

**Table 5:** Top 15 most frequently mentioned words related to sleep issues among users with attention-deficit/hyperactivity disorder (ADHD) and users without ADHD.

ADHD group	Non-ADHD group
Melatonin	Alarm
Groggy	Awake
Deprived	Sleep
Sleep	Snooze
Restless	Wide
Lesser	Snoozebutton
Asleep	Solution
Dive	Fluffiness
Exhausted	Yawn
Awake	Ladybird
Insomnia	Asleep
Anxious	Wake
Gummies	Rested
Waking	Laying
Severely	Clock

#### Topic 4: Drug Abuse

As shown in Table 6, drugs are often associated with addiction in ADHD data sets. Some users with ADHD mentioned that they had problems with drug abuse:

...I’m a drug addict you’re my favorite poison.

...When is the right time to tell them that I’m a drug addict.

Adderall is the primary treatment medicine for patients with ADHD, which gives the feeling of euphoria and confidence to help people with ADHD get focused and concentrated, and some patients even use it to lose weight owing to its side effects of appetite suppression [40]. Therefore, some users with ADHD abuse Adderall by taking more than their doctors prescribed. A user with ADHD complained that they could not get enough Adderall to mitigate their symptoms:

...Idk what I am going to do if my doctor can’t prescribe me more of my meds. The fact that I NEED this medication just to be able to function as a somewhat normal human being, but they treat me like a freaking drug addict because dumbasses took advantage.

For some users with ADHD, when they no longer feel satisfied with the effects Adderall has on them, they turn to other drugs, such as nicotine, weed, and methamphetamine (meth), to obtain the desired effect:

...Postmating a vape for lunch is the most adderall thing I’ve ever done.

...Adderall isn’t working anymore just give me actual meth.

In the non-ADHD data set, some users enjoyed taking drugs, mainly weed and nicotine. However, other users without ADHD discussed the harmful effects of drug use and smoking. We selected several common drugs, such as meth, weed, nicotine, heroin, and cocaine, and counted their occurrences in each group of tweets, as shown in Table 7. Overall, users without ADHD talked about drugs less frequently than users with ADHD. The whole word list correlated to this topic is shown in Multimedia Appendix 1.

**Table 6:** Top 15 most frequently mentioned words related to drug abuse among users with attention-deficit/hyperactivity disorder (ADHD) and users without ADHD.

ADHD group	Non-ADHD group
Weed	Smoking
Meth^a^	Weed
Adderall	Vaping
Addicted	Smoke
Cocaine	Nicotine
Overdose	Smoker
Addiction	Cigarette
Nicotine	Blunt
Rue	Reduction
Drug	Health
Cigarette	Tobacco
Sober	Harmful
Addict	Risk
Smoking	Harm
Vape	Safer

^a^Meth: methamphetamine.

**Table 7:** Occurrence counts of drugs in the attention-deficit/hyperactivity disorder (ADHD) and non-ADHD data sets.

Drugs	Meth^a^	Nicotine	Weed	Heroin	Cocaine	Caffeine	Adderall
Count in the ADHD group	597	423	7130	359	747	1749	1805
Count in the non-ADHD group	247	436	3080	230	452	351	117

^a^Meth: methamphetamine.

### Sentiment Classification

#### Frequency

**Figure 2:**
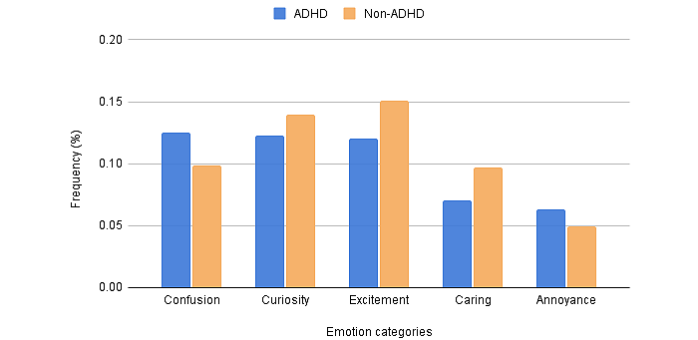
Average frequency distributions of emotion categories. ADHD: attention-deficit/hyperactivity disorder.

In this study, frequency refers to how often people experience emotions. For each user, we calculated the frequency of each emotion based on the number of tweets categorized by emotion as a percentage of the total number of tweets. Of the 27 emotion categories based on the model, the 5 most common emotions that users with ADHD expressed were confusion (ADHD: 12.54%; non-ADHD: 9.84%), curiosity (ADHD: 12.30%; non-ADHD: 13.93%), excitement (ADHD: 12.02%; non-ADHD: 15.09%), caring (ADHD: 7.08%; non-ADHD: 9.66%), and annoyance (ADHD: 6.35%; non-ADHD: 4.91%). Compared with users without ADHD, users with ADHD had a higher average emotion frequency for confusion and annoyance and a lower average frequency for curiosity, excitement, and caring. All 5 emotions were expressed significantly differently by users with ADHD and users without ADHD with *P*<.001. The average frequencies of the 5 emotion categories are shown in Figure 2.

#### Intensity

**Figure 3:**
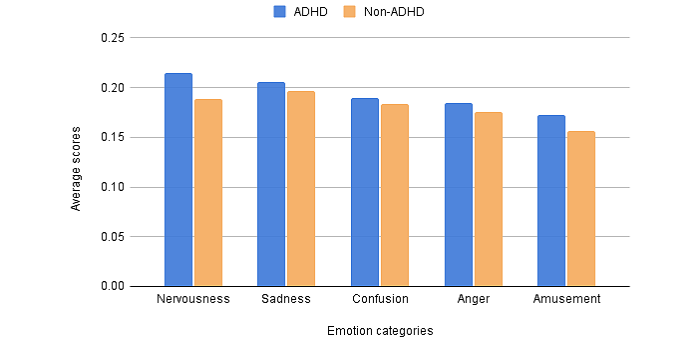
Average scores of emotion categories. ADHD: attention-deficit/hyperactivity disorder.

For each emotion category, a higher average score from the sentiment model represents more intense feelings of that emotion. The 5 emotions with the highest average scores in the ADHD and non-ADHD data sets were nervousness (ADHD: 0.215; non-ADHD: 0.188), sadness (ADHD: 0.205; non-ADHD: 0.196), confusion (ADHD: 0.190; non-ADHD: 0.184), anger (ADHD: 0.185; non-ADHD: 0.175), and amusement (ADHD: 0.172; non-ADHD: 0.156). All 5 emotion categories were expressed significantly differently (*P*<.001). Figure 3 shows that the average score of each emotion category of users with ADHD was higher than that of the control group, indicating that users with ADHD were more sensitive to emotions and felt them more intensively.

### Twitter Interaction Features

#### Posting Hour and Posting Day

**Figure 4:**
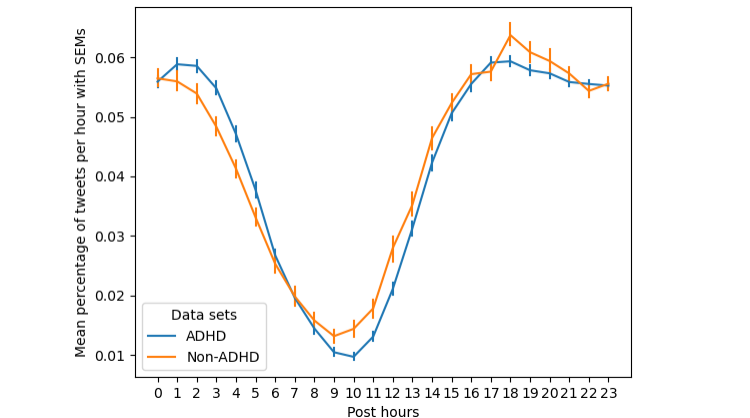
Percentage of posted tweets per hour. ADHD: attention-deficit/hyperactivity disorder; SEM: SE of mean.

**Figure 5:**
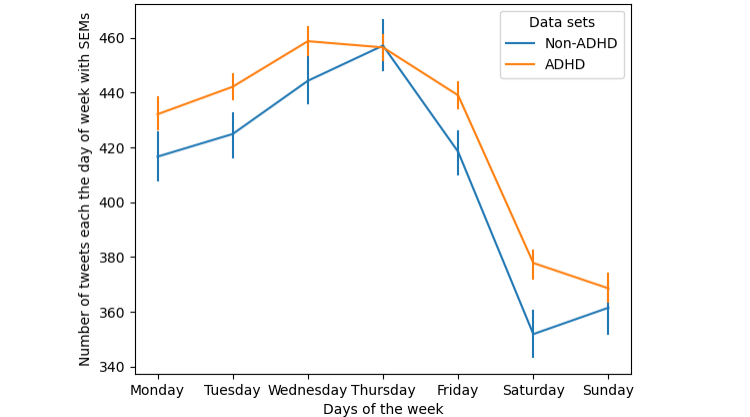
The number of posted tweets per day. ADHD: attention-deficit/hyperactivity disorder; SEM: SE of mean.

We calculated the number of tweets posted per hour as the percentage of all tweets for each user in the ADHD and non-ADHD data sets. Figure 4 shows that users with ADHD are more active on Twitter than users without ADHD during the night, from 12 AM to 6 AM. The interactions between the data sets factor and posthour factor were measured using 2-way repeated-measures ANOVA (*P*<.001). The number of tweets posted per day throughout the week is compared in Figure 5 (*P*=.04). Overall, users with ADHD were more active and posted more tweets daily throughout the week.

#### Original Ratio and Retweeted Tweet Ratio

**Figure 6:**
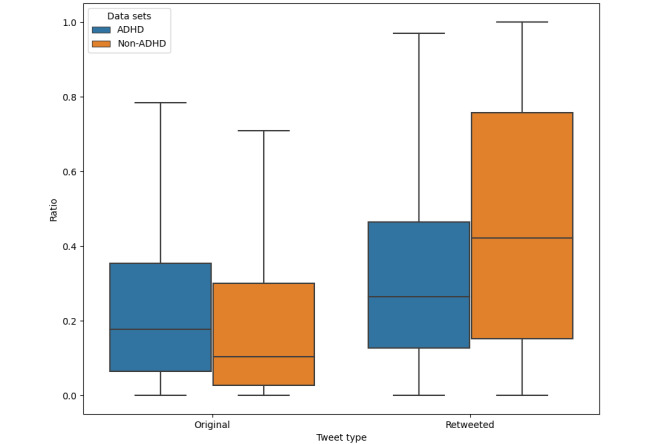
Boxplot of original ratio and retweet ratio in attention-deficit/hyperactivity disorder (ADHD) and non-ADHD data sets.

Tweets were classified into 4 types: original, retweeted, replied, and quoted tweets. We compared the ratios of original tweets and the ratios of retweeted tweets in the ADHD and non-ADHD data sets and examined the average values and distributions of these 2 ratios. The results are shown in Figure 6. The average ratio of original tweets for users with ADHD and users without ADHD were 28.48% and 19.52%, respectively. The average ratio of retweeted tweets for users with ADHD and users without ADHD was 31.35% and 45.80%, respectively. There were significant differences between the 2 data sets for the original tweet ratio (*P*<.001) and retweeted tweet ratio (*P*<.001) by applying the Mann-Whitney *U* test.

#### Followers and Followings

**Figure 7:**
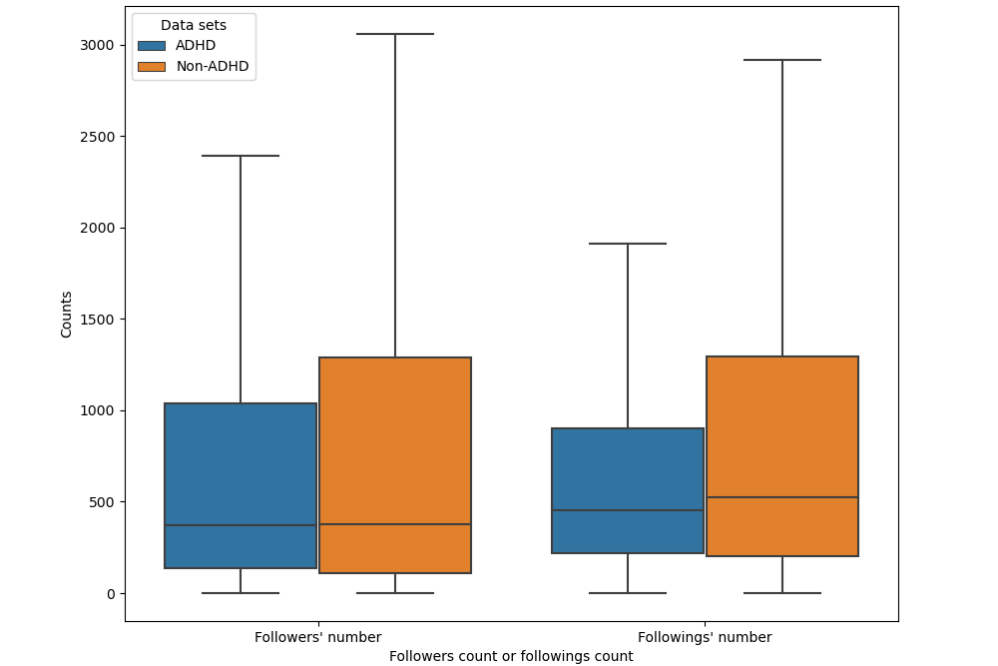
Boxplot of the number of followings and followers in attention-deficit/hyperactivity disorder (ADHD) and non-ADHD data sets.

There was a significant difference in the number of followings between the 2 data sets (*P*<.001). The average number of followings for users with ADHD was 804, whereas the average number of followings for users without ADHD was 1323. The difference in the number of followers was not statistically significant (*P*=.06). Figure 7 presents a boxplot of the number of followers and the followings.

## Discussion

### Principal Findings

In this study, we examined the web-based behaviors and interactions of self-reported users with ADHD with a control group of users without ADHD on Twitter. The results suggested that users with ADHD described themselves differently under the topics of concentration ability, time management, sleep issues, and drug abuse compared with users without ADHD. Furthermore, the frequency and intensity of various emotions expressed differed notably. We also found a considerable difference in tweet activities between users with ADHD and users without ADHD in terms of the number of tweets posted, the type of tweets posted, and the number of followings. Given the statistic that 93.4% of Twitter users are aged ≥18 years [9] and the evidence that symptoms of ADHD may vary between children and adults [6], these findings could help researchers and clinicians improve the diagnostic criteria for adult ADHD and develop social media as a new, objective complementary approach to screening and diagnosing ADHD.

The findings of this study corroborate previous findings regarding the symptoms of adult ADHD. Regarding concentration ability and time management, users with ADHD often mentioned difficulty sustaining attention, getting distracted easily, and failing to prioritize tasks and activities, which the DSM-5 lists as typical symptoms of inattention [5]. Hyperfocus, another prevalent clinical behavior in adults with ADHD [41], was found in tweets of users with ADHD. However, hyperfocus is not included in the current ADHD diagnostic criteria of DSM-5. Consistent with previous findings of the association between sleep disturbance and ADHD symptoms [42-44], the topic of sleep issues revealed that users with ADHD experienced restlessness and insomnia, causing them to feel physical and mental exhaustion. ADHD has long been recognized as a risk factor for abuse of prescription drugs, such as Adderall and other licit or illicit drugs, such as weed, caffeine, and meth [45-48]. In accordance with this finding, users with ADHD tweeted about their use of drugs to manage symptoms that led to addiction and abuse.

The sentiment analysis results showed that users with ADHD expressed emotions differently than users without ADHD. Users with ADHD felt negative emotions, such as confusion and annoyance, more frequently than users without ADHD and maintained more intense feelings overall for all emotions. This could be due to emotional dysregulation, one of the common symptoms reported by adult patients with ADHD [49]. One comprehensive review concluded that 34% to 70% of adults with ADHD have emotional dysregulation, compared with 25% to 45% of children with ADHD [50]. Patients with ADHD with emotional dysregulation cannot control emotions, often burst out of anger, shift moods suddenly, and amplify frustration and annoyance with minor stimulations [51,52]. Being hypersensitive and easily affected by negative feelings makes people with ADHD feel nervous and confused because they may not realize that their inability to moderate emotions is due to ADHD and cannot find a way to manage it [49].

Another finding was that users with ADHD felt excitement less often than those without ADHD, which indicates a boredom proneness among users with ADHD. Boredom proneness has been considered highly correlated with impaired attention [53-55]. Adult patients with ADHD with boredom proneness often behave poorly in sustaining attention and show more severe symptoms of ADHD [55,56]. For users with ADHD on Twitter, boredom might be triggered by intensive internet use by young adults [57,58]. One unanticipated finding is that users with ADHD expressed curiosity less frequently on Twitter than those without ADHD. It has been recognized that people with ADHD are more curious and regularly seek new and exciting news. A possible explanation for our finding is due to the skewed distribution. The distribution of curiosity in the non-ADHD data set is skewed left, so the average frequency of curiosity in the non-ADHD data set may not refer to the actual average frequency for all non-ADHD populations. Another possible explanation is that the curiosity of users with ADHD is not recorded on Twitter because they are easily distracted by another topic before tweeting about their current curiosity. We acknowledge that there may be a discrepancy between the manifestations of ADHD symptoms on Twitter and offline; therefore, further studies should be conducted.

This study found that users with ADHD tweeted more at night between midnight and 6 AM, whereas they tweeted less during the daytime compared with users without ADHD. This finding may correspond to the sleep issues, restlessness, and insomnia uncovered in the topic modeling analysis of users with ADHD. According to the posting pattern by day throughout the week, the daily posting activity trends of users with ADHD and users without ADHD are vastly similar, but users with ADHD kept more active and posted more tweets each day. Users with ADHD posted more original tweets than users without ADHD. These 2 patterns may be related to the hyperactivity and impulsivity symptoms of ADHD. Patients with ADHD usually talk excessively [5], leading to frequent expressions on social media. Furthermore, as mentioned earlier, boredom proneness may lead to internet addiction and is a possible reason why users with ADHD are more active and addicted to Twitter. Although users with ADHD tweeted more on Twitter, they followed fewer users on average than did users without ADHD, suggesting that they were more self-centered. This could also be due to the rapid shift in attention of users with ADHD. However, more examination is needed.

### Limitations

There are several limitations to our study. First, we selected users with ADHD using specific keywords related to their self-reported patient experiences. Without proof of clinical assessment, we cannot determine whether users were professionally diagnosed with ADHD and thus rely on self-reporting. In the same manner, a potential uncontrolled factor in which users who do not explicitly self-report having ADHD may have resulted in the non-ADHD data set including users with ADHD. Second, although the results provided evidence that users with ADHD manifested different patterns compared with users without ADHD, such as affected by sleep disturbance and drug abuse, we cannot ignore that they may be caused by comorbid disorders that co-occur with ADHD, such as depression and anxiety disorders. Third, ADHD is complex, and its diagnosis is subjective and lacks standardization [59]. The interpretation of research findings regarding their topics, sentiment analysis, and tweet interactions contained a degree of conjecture based on empirical and clinical experience and may not fully reflect the full picture of people with ADHD. Finally, the analysis was conducted after eliminating emojis during the data cleaning process because of the multitude connotations of emojis.

### Future Directions

This study provides a preliminary examination of the behavioral and interaction patterns of users with ADHD on Twitter. Twitter can be a prospective and advantageous tool to help improve diagnostic criteria and detect users with ADHD based on their historical content on social media. It is necessary to further compare the commonalities and differences between users with ADHD on social media and medically diagnosed patients with ADHD to integrate social media and clinical assessments to screen and diagnose ADHD. Considering that the symptoms of ADHD may vary with gender, age, and other characteristics, a future study is needed to extract the demographics of users with ADHD on Twitter to observe the differences and changes in different subgroups. In addition, combining image and emoji analysis in tweets with text analysis may provide multidimensional and further insights into the behavioral and interaction patterns of users with ADHD. Given the impact that social media can have on people, it is also interesting to explore the positive and negative effects of social media on users with ADHD offline, and the relationship between social media and symptoms of ADHD.

### Conclusions

In summary, with the popularity of social media, an increasing number of people are sharing their thoughts, opinions, and feelings instantaneously on platforms such as Twitter, allowing for research on the reflection of mental health. By analyzing the text content, sentiment, and tweet interactions of users who disclosed their ADHD, our study found how users with ADHD express themselves on social media. The findings of this study corroborate the existing research on ADHD and provide new insights for further research. Our findings can help gain a deeper understanding of the thoughts, feelings, and behavior behind ADHD symptoms, and suggest that Twitter could serve as a powerful platform for improving ADHD diagnostic criteria, automating detection, and monitoring patients with ADHD.

## Appendix

Multimedia Appendix 1 Topic Analysis: searching keywords; frequently mentioned words under topics.

## Abbreviations

ADHD: attention-deficit/hyperactivity disorder
CDC: Centers for Disease Control and Prevention
DSM-5: Diagnostic and Statistical Manual of Mental Disorders, Fifth Edition
Meth: methamphetamine
NLTK: Natural Language Toolkit
PTSD: posttraumatic stress disorder

## Data Availability

According to Twitter’s developer agreement and policy [37], Twitter restricts the redistribution of Twitter content to third parties and only allows the sharing of Tweet IDs and User IDs for noncommercial research purposes. The Tweet IDs and User IDs used in this study will be available upon request from the authors.
