# Supplementary material for: Exploring the Behavior of Users With Attention-Deficit/Hyperactivity Disorder on Twitter: Comparative Analysis of Tweet Content and User Interactions
Source: J Med Internet Res. 2023 May 17;25:e43439. doi: 10.2196/43439 (PMC10233432; doi:10.2196/43439)
Supplement: Multimedia Appendix 1 [file jmir_v25i1e43439_app1.docx]

## Multimedia Appendix

# **Exploring ADHD Users’ Behavior on Twitter: A comparative Analysis of Tweet Content and User Interactions**

Liuliu Chen, Jiwon Jeong, Bridgette Simpkins, Emilio Ferrara

This file contains additional tables for topic analysis on ADHD and non-ADHD datasets.

Table 1. Keywords used for searching each topic on the topic model.

| Topic | Keywords used for search |
| --- | --- |
| Concentration ability | Focus, focused, task, attention, productive, concentration, concentrate, distraction |
| Time management | Priority, prioritize, organize, organizing, planning, plan, multitask, time management, decision-making |
| Sleeping issues | Sleep, insomnia, restless, awake, sleepy, wake up, chronic |
| Drug abuse | Drug, addiction, meth, smoking, smoke, nicotine, drug addiction, substance abuse, drug abuse |

Table 2. Top 50 most frequently mentioned words related to concentration abilities among users with ADHD and users without ADHD.

| ADHD group | Non-ADHD group |
| --- | --- |
| Productive | Attention |
| Focusing | Focused |
| Distracted | Focusing |
| Exhausted | Paying |
| Focus | Hollywood |
| Fixation | Teruyama |
| Symptom | Geometry |
| Fatigue | Listenlive |
| Mindfulness | Role |
| Motivation | Friends |
| Hyperfixation | Focus |
| Distraction | Impress |
| Fixated | Cityofmoncton |
| Struggle | Ancestry |
| Task | Span |
| Focused | Paranormal |
| Fixate | Iammissapril |
| Masquerade | Businesslogo |
| Chore | Diet |
| Selflove | Writingcommunity |
| Oct | Alyosha |
| Med | Flashbackfriday |
| Anxious | Familytree |
| Migraine | Harrystyles |
| Clyde | Vehicular |
| Napped | Bloggerstribe |
| Holistic | Dylanstatham |
| Insomnia | Chiddickstree |
| Neurodiversity | Familyhistory |
| Aware | Prabhas |
| Adderall | Newbrunswick |
| Showered | Netneutrality |
| Hyperfocus | Warden |
| Tired | Manslaughter |
| Difficult | Technologist |
| Bmi | Replying |
| Daunting | Raypeeest |
| Hyperfixations | Hamel |
| Depression | Akane |
| Learning | Rinarina |
| Bonnie | Schrader |
| Anxiety | Helpful |
| Coping | Madly |
| Crave | Fitness |
| Productivity | Solutionni |
| Process | Staups |
| Stimulation | Blogging |
| Adhd | Persfinguru |
| Mindful | Strategic |
| Overwhelm | Connection |

Table 3. Top 50 most frequently mentioned words related to time management among users with ADHD and users without ADHD.

| Adhd | Non-adhd |
| --- | --- |
| Prioritize | Ballot |
| Organize | Workshop |
| Organized | Folder |
| Dysfunction | Voter |
| Weakness | Election |
| Unmotivated | Linked |
| Organizing | Fujimoto |
| Task | Learning |
| Priority | Files |
| Strength | Voting |
| Clean | Organizing |
| Performative | Booklet_design |
| Resource | Vote |
| Workspace | Theatre |
| Hard | Document |
| Function | Republican |
| Mcconnell | Academic |
| Cleaning | Valued |
| Prioritizing | Fraud |
| Functionality | Storytelling |
| Chore | Classicfirearm |
| Understanding | Democrat |
| Folder | Joseline |
| Laundry | Corporatebrochure |
| Activist | Voted |
| Gossipgirlreboot | Tuned |
| Productive | Culy |
| Communication | Candidate |
| Tool | Carletonu |
| Challenging | Entered |
| Ability | Gop |
| Creativity | Edt |
| Abba | Businessflyer |
| Shovel | Democracy |
| Difficult | Rina |
| Stuff | Sweepstakes |
| Capable | Dems |
| Pantry | Course |
| Wellbeing | Rifle |
| Perfectionist | Polling |
| List | Interesting |
| Lack | Scope |
| Caregiver | Senate |
| Ocd | Architecture |
| Disability | Data |
| Akaashi | Trump |
| Drawer | Paicipation |
| Bipoc | Businessflyers |
| Manage | Mail |
| Bookshelf | Selfcare |

Table 4. Top 50 most frequently mentioned words related to sleep issues among users with ADHD and users without ADHD.

| ADHD group | Non-ADHD group |
| --- | --- |
| Melatonin | Alarm |
| Groggy | Awake |
| Deprived | Sleep |
| Sleep | Snooze |
| Restless | Wide |
| Lesser | Snoozebutton |
| Asleep | Solution |
| Dive | Fluffiness |
| Exhausted | Yawn |
| Awake | Ladybird |
| Insomnia | Asleep |
| Anxious | Wake |
| Gummies | Rested |
| Waking | Laying |
| Severely | Clock |
| Deep | Bed |
| Wake | Exhausted |
| Pill | Routine |
| Sleeping | Elsword |
| Tired | Sunrise |
| Alarm | Eyed |
| Woke | Rushed |
| Sleepy | Confusion |
| Eff | Easier |
| Nap | Yggdrasil |
| Wana | Yoga |
| Med | Spent |
| Gummy | Melatonin |
| Distressed | Fully |
| Noon | Night |
| Slept | Yawning |
| Benadryl | Accept |
| Deprivation | Insomnia |
| Woken | Helleen |
| Hr | Productivity |
| Morning | Pondered |
| Overload | Improve |
| Bed | Frazzled |
| Depressed | Sleepy |
| Headache | Enhance |
| Evil | Impaled |
| Supplement | Nap |
| Yawning | Slept |
| Tylenol | Method |
| Mechanism | Stay |
| Cbd | Heynow |
| Delirious | Crawling |
| Cycle | Reduce |
| Caffeine | Mon |
| Stressed | Alyosha |

Table 5. Top 50 most frequently mentioned words related to drug abuse among users with ADHD and users without ADHD.

| ADHD group | Non-ADHD group |
| --- | --- |
| Weed | Smoking |
| Meth | Weed |
| Adderall | Vaping |
| Addicted | Smoke |
| Cocaine | Nicotine |
| Overdose | Smoker |
| Addiction | Cigarette |
| Nicotine | Blunt |
| Rue | Reduction |
| Drug | Health |
| Cigarette | Tobacco |
| Sober | Harmful |
| Addict | Risk |
| Smoking | Harm |
| Vape | Safer |
| Heroin | Shit |
| Dealer | Vape |
| Vaping | Mf |
| Quiz | Safe |
| Stimulant | Hookah |
| Smoke | Trippin |
| Sobriety | Blunts |
| Caffeine | Cigar |
| Smoker | Ima |
| Cigs | Health |
| Rehab | Lmaoo |
| Prescribed | Alternative |
| Alcohol | Smokin |
| Theythem | Approach |
| Quitting | Ganja |
| Management | Liquor |
| Adhd | Fda |
| Fentanyl | Liver |
| Jules | Iont |
| Duolingo | Study |
| Euphoria | Drinking |
| Marijuana | Kkundrrasquad |
| Appetite | Reducing |
| Substance | Mimosa |
| Med | Government |
| Pronoun | Sipping |
| Tutor | Wit |
| Coke | Policy |
| Overdose | Elsword |
| Smoked | Scientific |
| Ritalin | Dtnt |
| Pill | Fenna |
| She | Product |
| Tobacco | Coulda |
| Ketamine | Disease |
